# Long-term longitudinal evaluation of the prevalence of SARS-CoV-2 antibodies in healthcare and university workers

**DOI:** 10.1038/s41598-022-09215-8

**Published:** 2022-03-25

**Authors:** Pascale Huynen, Céline Grégoire, Stéphanie Gofflot, Laurence Seidel, Nathalie Maes, Laura Vranken, Sandra Delcour, Michel Moutschen, Marie-Pierre Hayette, Philippe Kolh, Pierrette Melin, Yves Beguin

**Affiliations:** 1 Division of Medical Microbiology, Unilab, CHU of Liège, Liège, Belgium; 2 Center for Interdisciplinary Research on Medicines, University of Liège, Liège, Belgium; 3 Department of Hematology, CHU of Liège, Avenue de l’hôpital 1, 4000 Liège, Belgium; 4 Biothèque Hospitalo-Universitaire de Liège (BHUL), CHU of Liège, Liège, Belgium; 5 Department of Biostatistics and Medico-Economic Information, CHU of Liège, Liège, Belgium; 6 Unilab, CHU of Liège, Liège, Belgium; 7 Division of Infectious Diseases and General Internal Medicine, CHU of Liège, Liège, Belgium; 8 GIGA-I3 Laboratory of Immunology, University of Liège, Liège, Belgium; 9 Department of Information System Management, CHU of Liège, Liège, Belgium; 10 Department of Biomedical and Preclinical Sciences, University of Liège, Liège, Belgium; 11 GIGA-I3 Laboratory of Hematology, University of Liège, Liège, Belgium

**Keywords:** Viral infection, Epidemiology, Infectious-disease diagnostics

## Abstract

Asymptomatic and pauci-symptomatic cases contribute to underestimating the prevalence of severe acute respiratory syndrome coronavirus 2 (SARS-CoV-2) infections. Moreover, we have few studies available on the longitudinal follow-up of SARS-CoV-2 antibodies after natural infection. We tested staff members of a Belgian tertiary academic hospital for SARS-CoV-2 IgG, IgM, and IgA antibodies. We analyzed the evolution of IgM and IgG after 6 weeks, and the persistence of IgG after 3 and 10 months. At the first evaluation, 409/3776 (10.8%) participants had a positive SARS-CoV-2 serology. Among initially seropositive participants who completed phases 2 and 3, IgM were still detected after 6 weeks in 53.1% and IgG persisted at 12 weeks in 82.0% (97.5% of those with more than borderline titers). IgG levels were higher and increased over time in symptomatic but were lower and stable in asymptomatic participants. After 10 months, 88.5% of participants had sustained IgG levels (97.0% of those with more than borderline titers).

## Introduction

Belgium is one of the countries most severely affected by the severe acute respiratory syndrome coronavirus 2 (SARS-CoV-2) causing COVID-19 (COronaVIrus Disease 2019)^1^, with nearly 67,000 confirmed cases and 1900 deaths per million population on March 1st, 2021. However, these figures underestimate the true cumulative incidence in Belgium given the shortage of reverse transcription polymerase chain reaction (RT-PCR) tests during the first wave and the existence of asymptomatic subjects.

The reference method for diagnosing COVID-19 infection is the genomic detection of SARS-CoV-2 specific sequences by RT-PCR on respiratory specimens, while serological tests detecting antibodies against SARS-CoV-2 specific epitopes allow diagnosis at a later stage or retrospectively^2^. Depending on studies and methods used, seroconversion has been reported as soon as a few days after onset of symptoms with IgM preceding IgG, or within 2–3 weeks with IgM and IgG arising simultaneously^3,4^. Higher IgG levels are observed in symptomatic than in pauci-/asymptomatic patients^5^.

Many epidemiological studies have been performed in the general population or among healthcare workers, but information is lacking on the serologic evolution in these populations. Through four-phase monitoring of the humoral response to SARS-CoV-2 infection in nearly 4000 volunteer healthcare and non-healthcare workers, this study aims to (A) evaluate the incidence of pauci/asymptomatic SARS-CoV-2 infections during the first wave in this active adult population, and (B) evaluate short- and long-term persistence of SARS-CoV-2 IgG antibodies.

## Results

### Phase 1

**Figure 1:**
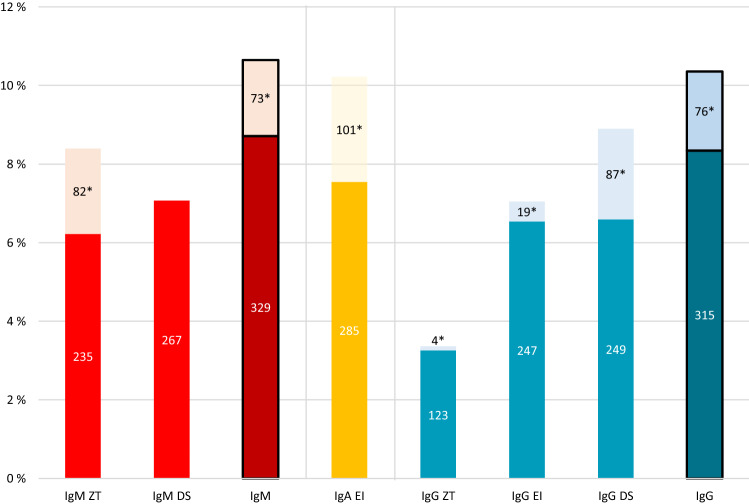
SARS-CoV-2 seroprevalence (%) in phase 1 according to the type of test used. * indicates borderline results. IgM: positive for ≥ 1 IgM test; IgG: positive for ≥ 1 IgG test. *DS* DiaSorin, *EI* EuroImmun, *ZT* ZenTech.

Between April 6 and May 5 (only 14 tested beyond April 30) 2020, 3776 staff members were tested. Median age was 39.8 years (range 20.1–81.3), and 74.7% were women. In order to decide which tests were more performant and suitable for longitudinal follow-up, we initially tested SARS-CoV-2 seropositivity with the IgM ZenTech, IgM DiaSorin, IgA EuroImmun, IgG ZenTech, IgG DiaSorin and IgG EuroImmun tests, which yielded seropositivity rates of 8.4%, 7.1%, 10.2%, 3.4%, 8.9%, and 7.0%, respectively (Fig. 1). Overall, 402 (10.6%), 386 (10.2%) and 391 (10.4%) participants raised a detectable IgM (ZenTech and/or DiaSorin), IgA (EuroImmun) and IgG (Zentech and/or EuroImmun and/or DiaSorin) immune response against SARS-CoV-2, respectively. Concordance was 94.2% between DiaSorin and ZenTech IgM tests and 95.5% between DiaSorin and EuroImmun IgG tests. For the subsequent phases of the study, we selected the more sensitive IgM ZenTech (phase 2) and IgG DiaSorin (phases 2, 3 and 4) tests.

### Phases 2 and 3

We analyzed the evolution of IgM (using the ZenTech test) 6 weeks after the first measurement (phase 2) and IgG (using the DiaSorin test) after 6 (phase 2) and 12 weeks (phase 3). Among the initial 3776 subjects, 3187 and 2498 subjects completed phases 2 and 3, respectively.

IgM seropositivity decreased from 317/3776 (8.4%) at baseline to 232/3187 (7.3%) at phase 2 (p = 0.013). Among 286 initially seropositive participants also evaluated in phase 2, 134 became seronegative (46.9%) while 152 (53.1%) still had detectable IgM.

**Figure 2:**
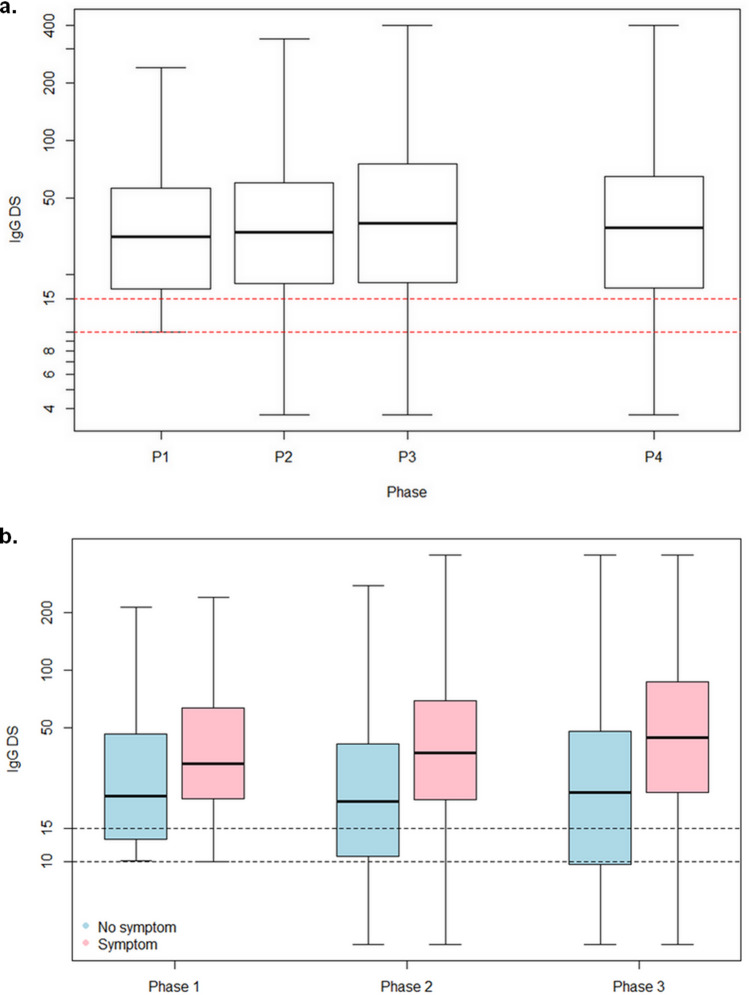
Evolution of IgG levels in seropositive participants at phase 1 (by the DiaSorin test) between phases 1, 2, 3 and 4. (**a**) Evolution of IgG levels (by the DiaSorin test) of subjects who participated in the four phases. (**b**) Evolution of IgG levels of seropositive participants at phase 1 (by the DiaSorin IgG test) according to the presence or absence of symptoms during phase 1. Box-plots show median, interquartile range, and range.

IgG seropositivity by the Diasorin test was observed in 336/3776 (8.9%), 317/3187 (9.9%), and 288/2498 (11.5%) subjects in phase 1, 2, and 3, respectively. However, participation to phases 2 and 3 was significantly higher in phase 1 seropositive subjects (OR 2.28 [95% CI 1.73–2.99], p < 0.0001), representing a selection bias. Therefore, we analyzed separately the results of 2433 subjects who completed the 3 phases. Among them, seropositivity rates were 11.0% (267/2433), 11.8%, and 11.4% at phases 1, 2, and 3, respectively, which represents a significant increase only between phases 1 and 2 (p = 0.026 by general linear mixed model) (consistent with the low SARS-CoV-2 circulation in Belgium in the period between phases 2 and 3). Among the 200 participants with an IgG level ≥ 15 AU/mL in phase 1, only 4 became seronegative in phase 2 (among which 2 became borderline in phase 3) and 3 in phase 3. New IgG seroconversion occurred in 55/2155 participants (2.5%) in phase 2 (among which 47 (85.5%) remained positive in phase 3), and 8 participants (0.2%) in phase 3. In phase 1 seropositive patients, mean IgG levels by the Diasorin test were 44.4 ± 52.9 AU/mL in phase 1, 46.4 ± 54.6 AU/mL in phase 2, and 53.8 ± 64.9 AU/mL in phase 3, corresponding to a slight increase in phase 3 compared to phase 2 (p = 0.04) (Fig. 2a). IgG levels were higher in symptomatic than in asymptomatic participants in all 3 phases (p < 0.0001) (Fig. 2b). Asymptomatic subjects had stable IgG levels over phases 1, 2, and 3, while IgG levels increased over time in symptomatic subjects (p < 0.0001).

### Phase 4

Participants testing positive in phases 1, 2, and/or 3 for ≥ 1 IgG and/or for ≥ 2 IgM or IgA tests were again invited for IgG DiaSorin testing 10 months after the first sampling; 345 returned for this 4th phase. Among participants tested positive by the DiaSorin IgG test during phase 1, 230/256 (89.8%) remained positive in phase 4. In these 256 subjects, mean IgG levels were 47.2 ± 53.4 AU/mL in phase 1, 50.9 ± 58.5 AU/mL in phase 2, 59.0 ± 68.8 AU/mL in phase 3, and 54.1 ± 62.9 AU/mL in phase 4, and can be considered stable (p = 0.090) (Fig. 2a). Persisting IgG seropositivity represented 83.5% (288/345) of previously overall seropositive participants, 88.5% (277/313) of those IgG seropositive by the DiaSorin test at any previous phase, and 95.9% (164/171) of those IgG seropositive for both EuroImmun and DiaSorin IgG tests at phase 1. Most participants who became seronegative had borderline IgG levels (10–15 AU/mL) in phase 3. Indeed, among seropositive participants with IgG levels ≥ 15 AU/mL in phases 1, 2 and/or 3, 86.8% remained seropositive, 10.2% became borderline, and 3% seronegative in phase 4. Among those with only borderline IgG in phases 1, 2 and/or 3, 59.6% became seronegative, 17.0% remained borderline, and 23.4% became seropositive.

## Discussion

We initially used a variety of then available tests in order to identify which ones were more performant and suitable for longitudinal follow-up. By doing so, in the first study phase, i.e. 5–9 weeks after the first Belgian COVID-19 case, we report respective rates of SARS-CoV-2 IgM, IgA and IgG seropositivity of 10.6%, 10.2% and 10.4% among Belgian healthcare and non-healthcare workers. These rates were thus quite superimposable, indicating that we indeed probably captured the whole spectrum of seropositivity in our population. While a lower seroprevalence has been reported using rapid tests^6,7^, a similar rate of seropositivity was observed during the first wave of this pandemic in other high-prevalence regions^8–12^. In comparison, the cumulative incidence of COVID19 cases confirmed by RT-PCR was approximately 0.5% at that time in Belgium (51,858 confirmed cases for a population of 11,522,440), which underlines the substantial underestimation of the figures at the beginning of the pandemic. The combination of several tests might however increase the rate of false positivity, but these assays have demonstrated their excellent specificity (98.6–100% for the EuroImmun IgG assay and 99.3–100% for the DiaSorin IgG assay)^13–15^, which was confirmed in our validation tests using samples collected long before the COVID-19 pandemic. Concordance among similar Ig tests was very good and we therefore selected the most sensitive tests, i.e. the Zentech IgM and the Diasorin IgG tests, for our longitudinal study.

Indeed, we performed a longitudinal follow-up of S antibodies in nearly 2500 participants with samples collected 6 and 12 weeks after the first measurement. While specific IgM disappeared within 6 weeks in nearly half of the participants, IgG-S1/S2 were still detected in 87.2% and 82.0% of the initially seropositive participants after 6 and 12 weeks, respectively (these figures even increased to 98.0 and 97.5% when considering only participants with initial IgG levels > 15 AU/mL). These results are consistent with previous reports that antibodies against the S protein are durable^16,17^, and that the DiaSorin test is effective in detecting long-term persistent antibodies^18^. We further show here that the duration of such persistence may be longer than previously anticipated and higher among symptomatic participants with greater IgG titers. Indeed, we tested initially seropositive participants 10 months later and 83.5% (97% among those with IgG ≥ 15 AU/mL) remained positive and their mean antibody titer did not decrease, although some of them became borderline positive. This is in agreement with a smaller study with shorter follow-up identifying 81 (9.5%) seropositive participants among 850 healthcare workers from 17 Belgian hospitals, among whom 91% remained positive after 4 months^19^, as well as in a pediatric cohort (persisting IgG at 62 days in 45 seropositive patients)^20^. This is particularly important as IgG-S1/S2 correlate well with IgG neutralizing antibodies^21^ and appear to be protective against further active infection^22,23^. Hence, complete loss of anti-SARS-CoV2 IgG within 10 months occurs rarely in subjects with an IgG titer ≥ 15 AU/mL.

Our study has some limitations. Our participants are mostly young healthcare workers that may not be representative of the overall population. Nevertheless, the strength of this study is the use of several tests and the long-term longitudinal follow-up in a large population.

In conclusion, we demonstrate a high prevalence of SARS-CoV-2 seropositivity in a population of healthcare and non-healthcare workers in Belgium, including a number of asymptomatic persons, despite the (imperfect) protective measures and the lockdown that was in effect during the initial sampling period. We highlight the stability of IgG-S antibodies over time in most subjects. Whether this prolonged seropositivity confers protection against reinfections, especially with SARS-CoV-2 variants, is however unknown. As healthcare workers around the world are fighting against this pandemic, governments are trying to limit both casualties and the economic crisis, and vaccination campaigns are ongoing, this information is crucial to fully appreciate the health hazards and to adapt individual and collective protective measures against SARS-CoV-2.

## Methods

### Study design and participants

Workers of the University Hospital Center (CHU) of Liège across all sites and of the University of Liège (ULiège) on the Sart-Tilman site were invited to participate in the biobanking project coupled with the SARS-CoV-2 serologic testing at three time-points (days 0, 45, and 90), except when suspected of COVID-19 within 14 days before testing. From April 3 to May 5, 2020, 3776 participants registered for the first testing. We collected general information and personal health history. Participants testing positive in phases 1, 2, and/or 3 (positive for ≥ 1 IgG and/or for ≥ 2 IgM or IgA tests) were further invited to be tested for SARS-CoV-2 IgG 10 months after the first sampling.

This study was approved by the CHU of Liège Ethics Committee under number 2020/117 and has been performed in accordance with the Declaration of Helsinki. An informed consent form was signed by each participant. Data were recorded in a centralized database and pseudo-anonymized before statistical analysis.

### Serological testing

Samples were analyzed for specific IgA, IgM, and IgG against SARS-CoV-2 with three methods: a rapid immunochromatographic assay detecting IgM and IgG (QuickZen, ZenTech, Liège, Belgium), an Enzyme-Linked Immuno-Sorbent Assay (ELISA) detecting IgA and IgG (EuroImmun, Luebeck, Germany), and a ChemiLuminescent Immuno-Assay (CLIA) testing IgM and IgG (DiaSorin, Saluggia, Italy). All assays were performed according to manufacturers’ protocols. We then chose to analyze the evolution of IgM using the ZenTech test 6 weeks after the first measurement (phase 2) and IgG using the DiaSorin test after 6 weeks (phase 2), 12 weeks (phase 3) and 10 months (phase 4).

The QuickZen COVID-19 IgM/IgG immunochromatographic test uses colloidal gold-labelled recombinant antigens of the receptor binding domain (RBD) of the spike protein for qualitative detection of IgM and IgG^13^. Sensitivity was assessed on 251 consecutive samples from 68 hospitalized patients with RT-PCR-confirmed COVID-19. Sensitivities between days 0–6, 7–13, and after day 14 were 62.0%, 83.2%, and 98.6% for IgM, and 34.0%, 55.7%, and 94.2% for IgG, respectively. Specificity on 128 samples collected before July 2019 was 92.2% (95% CI 86.1–96.2%) and 99.2% (95% CI 95.7–99.9%) for IgM and IgG, respectively.

EuroImmun anti-SARS-CoV-2 IgA and IgG are semi-quantitative ELISA assays, using the S1 Spike protein, performed on the ETI-MAX®3000 analyzer (DiaSorin, Saluggia, Italy). According to the manufacturer’s instructions, ratios were interpreted as follows: < 0.8 negative, ≥ 0.8 and < 1.1 borderline, ≥ 1.1 positive. Performance was evaluated on samples of 150 COVID-19 and 100 control patients. By day 15 post-infection, 100% COVID-19 and 0% control patients had anti-SARS-CoV-2 IgA and IgG.

The LIAISON^®^SARS-CoV-2 S1/S2 IgM and IgG assays use a CLIA technology for quantitative determination of IgM against S1-RBD and IgG against S1 and S2 subunits of the Spike protein on the LIAISON^®^XL random access platform. Ratios were interpreted as follows: < 1.1 negative and ≥ 1.1 positive for IgM; < 10 AU/mL negative, ≥ 10 and < 15 AU/mL borderline, ≥ 15 AU/mL positive for IgG. Sensitivity of the IgG assay, assessed on 365 consecutive samples collected at diagnosis and up to 38 days thereafter from 82 hospitalized COVID-19 patients, was 30.6%, 70.2%, and 100%, between days 0–4, 5–14, and after day 15, respectively. Using a cut-off of 10 AU/mL for borderline values^24^ increases sensitivity without decreasing specificity (97.9%, evaluated on 147 samples collected before July 2019).

### Statistical analyses

General linear mixed model was used to study the evolution of IgG levels (in logarithm) with respect to phases. Calculations were done using SAS version 9.4, and figures were generated using R version 3.6.1. Results are considered significant at the 5% level (p < 0.05).

## Data Availability

The study protocol and individual participant data that underlie the results reported in this article, after de-identification, can be shared with investigators whose proposed use of the data has been approved by the ethic committee of the University Hospital of Liège. Data can be provided for meta-analysis or other projects comparing the seroprevalence estimates in different regions. Requests should be addressed to the senior author at yves.beguin@chuliege.be.
